# Metabolomics study of APETx2 post-conditioning on myocardial ischemia-reperfusion injury

**DOI:** 10.3389/fphar.2024.1470142

**Published:** 2024-12-06

**Authors:** Jing Li, Yiyong Wei, Yi Wang, Yue Zhang, Ying Xu, Huanhuan Ma, Lulin Ma, Qingfan Zeng

**Affiliations:** ^1^ Department of Anesthesiology, The Affiliated Baiyun Hospital of Guizhou Medical University, Guiyang, Guizhou Province, China; ^2^ School of Anesthesiology, Guizhou Medical University, Guiyang, Guizhou Province, China; ^3^ School of Anesthesiology, Zunyi Medical University, Zunyi, Guizhou Province, China; ^4^ Department of Anesthesiology, Affiliated Shenzhen Women and Children’s Hospital (Longgang) of Shantou University Medical College (Longgang District Maternity & Child Healthcare Hospital of Shenzhen City), Shenzhen, Guangdong Province, China; ^5^ Department of Oncology, The Second Affiliated Hospital of Zunyi Medical University, Zunyi, Guizhou Province, China; ^6^ Department of Anesthesiology, West China Hospital of Sichuan University, Chengdu, Sichuan Province, China

**Keywords:** ASIC3, miri, metabolites, APETx2 post-conditioning, phospholipid metabolites

## Abstract

**Background:**

Acid-sensing ion channels are activated during myocardial ischemia and are implicated in the mechanism of myocardial ischemia-reperfusion injury (MIRI). Acid-sensing ion channel 3 (ASIC3), the most pH-sensitive member of the ASIC family, is highly expressed in myocardial tissues. However, the role of ASIC3 in MIRI and its precise effects on the myocardial metabolome remain unclear. These unknowns might be related to the cardioprotective effects observed with APETx2 post-conditioning.

**Method:**

Rat hearts subjected to Langendorff perfusion were randomly assigned to the normal (Nor) group, ischemia/reperfusion (I/R) group, ASIC3 blockade (AP) group. Rat hearts in group AP were treated with the ASIC3-specific inhibitor APETx2 (630 nM). Molecular and morphological changes were observed to elucidate the role of ASIC3 in MIRI. Bioinformatics analyses identified differential metabolites and pathways associated with APETx2 post-conditioning.

**Results:**

APETx2 post-conditioning stabilized hemodynamics in the isolated rat heart model of MIRI. It also reduced myocardial infarct size, mitigated mitochondrial damage at the ultrastructural level, and improved markers of myocardial injury and oxidative stress. Further more, we observed that phosphatidylcholine, phosphatidylethanolamine, citric acid, cyanidin 5-O-beta-D-glucoside, and L-aspartic acid decreased after MIRI. The levels of these metabolites were partially restored by APETx2 post-conditioning. These metabolites are primarily involved in autophagy and endogenous cannabinoid signaling pathways.

**Conclusion:**

ASIC3 is potentially a key player in MIRI. APETx2 post-conditioning may improve MIRI through specific metabolic changes. This study provides valuable data for future research on the metabolic mechanisms underlying the effects of APETx2 post-conditioning in MIRI.

## Introduction

Coronary heart disease (CHD) is a common cardiovascular disorder among middle-aged and elderly individuals (Martin et al., 2024; Virani et al., 2023). Primary percutaneous coronary intervention (pPCI), thrombolytic therapy, and coronary artery bypass grafting enhance early reperfusion of ischemic myocardium. When facilities are available, pPCI is preferred over thrombolytic therapy for myocardial reperfusion (Byrne et al., 2023). However, these interventions invariably cause myocardial ischemia-reperfusion injury (MIRI) (Jneid et al., 2008). Research indicates that restoring blood flow to ischemic myocardium can result in myocardial damage, increased infarct size, and induced cardiomyocyte apoptosis (Martin et al., 2024). Therefore, understanding the process of MIRI and identifying targets for its prevention and therapy are of significant scientific and clinical importance.

In patients with acute myocardial infarction, the severity of metabolic acidosis is closely correlated with mortality rates (Martin et al., 2024; Jentzer et al., 2022; de Lucia et al., 2021). Specific cation channels in the myocardial cell membrane and associated organelles are involved in the pathophysiological processes of MIRI (Zhang H. N. et al., 2023; Malko and Jiang, 2020). At the cellular level, acidosis activates particular ion channels, including transient receptor potential (TRP) channels, sodium-hydrogen exchangers (NHE), and acid-sensing ion channels (ASICs). These channels are associated with energy metabolism disorders and calcium overload, ultimately leading to cardiomyocyte death (Chung et al., 2023; Koivisto et al., 2022; Redd et al., 2024).

ASICs, non-voltage-gated cation channels, are one of the five major subfamilies of the degenerin/epithelial sodium channel (DEG/ENaC) superfamily (Yoder et al., 2018). ASICs have seven subtypes, including ASIC1a, ASIC1b, ASIC1b2, ASIC2a, ASIC2b, ASIC3 and ASIC4. It is composed of four genes(ASIC1 to ASIC4) (Vullo and Kellenberger, 2020). The involvement of ASICs in the development of ischemia-reperfusion injury (IRI) has been well documented in the literature. Meredith A. Redd demonstrated that genetic ablation of ASIC1a improved cardiomyocyte viability in MIRI. Furthermore, applying a specific ASIC1a antagonist at the whole-organ level replicated this cardioprotective effect (Redd et al., 2021). Adaptive reperfusion following cerebral ischemia can suppress the production of ASIC3, thereby lowering Ca^2+^ inflow, preventing calcium overload, and further reducing cerebral IRI (Wang et al., 2013). Other studies have found that pH changes induced by myocardial ischemia can activate ASICs (Yang et al., 2023; Sutherland et al., 2001; Song et al., 2019). Meanwhile, ASICs are involved in acidosis-induced calcium overload in neurons and cardiomyocytes (Zuo et al., 2018). Among the ASIC family, ASIC3 is the most sensitive to variations in extracellular pH, with a half-maximal activation pH (pH50) of just 6.7^20^. It exists as a homopolymer in the heart (Dulai et al., 2021). Research has confirmed that ASIC3 is highly expressed in the human left ventricle (Redd et al., 2021). APETx2 is the most effective and selective ASIC3 inhibitor. It does not activate other ASIC subtypes (Diochot et al., 2004; Jalalvand et al., 2016).

In the present study, we hypothesized that APETx2 post-conditioning can improve cardiac function and affect principal metabolic pathways in hearts with MIRI. This study may provide new insights into the protective mechanism of APETx2 post-conditioning in MIRI.

## Materials and methods

### Animals

Twenty-four Sprague-Dawley (SD) rats (250–300 g, 16–20 weeks old) were housed in a specific pathogen-free (SPF) facility, maintained under a 12-h light/dark cycle with free access to food and water. Rats used in the study are not limited to either male or female. All experimental procedures were approved by the Animal Care and Use Committee of Zunyi Medical University (Zyfy-an-2023-0,147) and conducted following the Guide with arrive (Animal Research: Reporting of *In Vivo* Experiments) guidelines (Kilkenny et al., 2010).

### Perfusion protocol

**FIGURE 1:**
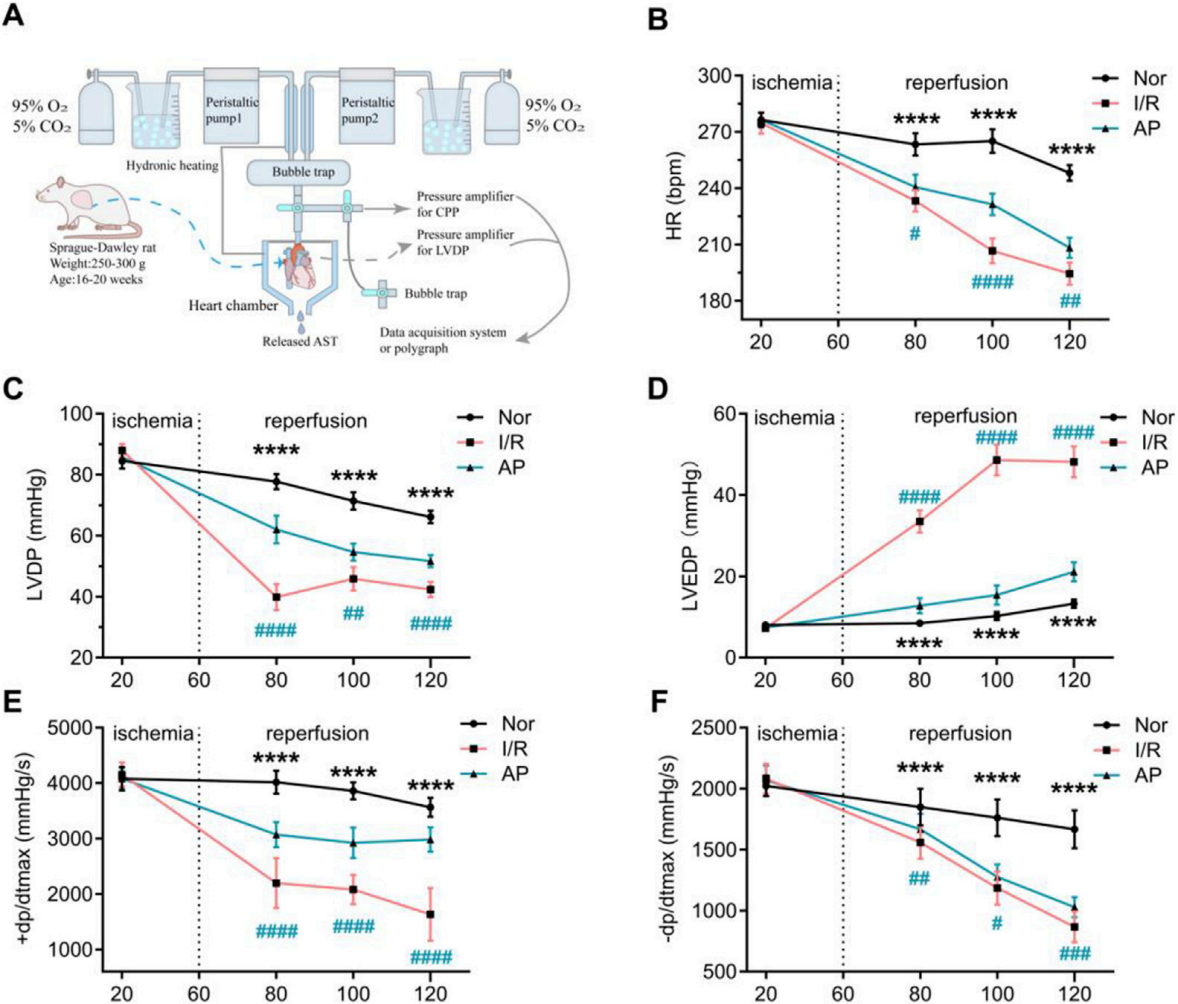
APETx2 post-conditioning stabilizes hemodynamic changes in MIRI hearts. **(A)** Schematic diagram of the isolated perfusion setup. **(B)** HR. As reperfusion time increased, HR in I/R rats decreased significantly. APETX2 post-conditioning slowed the HR decline (n = 8 per group). (C) LVDP. During MIRI, LVDP in I/R rats decreased significantly. APETx2 post-conditioning reduced the LVDP decline (n = 8 per group). (D) LVEDP. During reperfusion, LVEDP in I/R rats increased significantly, peaking at 55 mmHg. APETx2 post-conditioning reduced the LVEDP increase to an average of less than 20 mmHg (n = 8 per group). (E, F) ± dp/dtmax. During MIRI, +dp/dtmax in I/R rats decreased significantly. APETx2 post-conditioning mitigated this decline (n = 8 per group). One-way ANOVA, *P < 0.05; **P < 0.01; ***P < 0.001; ****P < 0.0001. All data are presented as mean ± SEM.

Rats received heparin (100 IU, i.p.) half an hour before anesthesia, which was induced with an intraperitoneal injection of sodium pentobarbital (40 mg/kg). The hearts were rapidly excised and placed in K-H solution (25.2 mM NaHCO_3_, 118.3 mM NaCl, 4.7 mM KCl, 1.18 mM MgSO_4_·7H_2_O, 1.70 mM CaCl_2_, 1.2 mM KH_2_PO_4_, and 11.1 mM glucose; 4°C, pH 7.40) (Rameshrad et al., 2021). The hearts were swiftly mounted on the Langendorff perfusion system (Prag et al., 2022). The schematic diagram of the perfusion apparatus is shown in Figure 1A. All hearts were equilibrated with K-H solution for 20 min to establish a uniform baseline. The rats were randomly assigned to three groups: normal group (Nor), ischemia-reperfusion group (I/R), and ASIC3 blockade group (AP) (n = 8 per group). Hearts in the group Nor were continuously perfused with K-H solution (38°C) for 120 min. Hearts in the group I/R were equilibrated with K-H solution for 20 min, followed by perfusion with St. Thomas’ solution (16.0 mM KCl, 110.0 mM NaCl, 16.0 mM MgCl_2_, 1.20 mM CaCl_2_, 10.0 mM NaHCO_3_, 4°C) until cardiac arrest. They were then subjected to global ischemia at 32°C for 40 min, followed by reperfusion with K-H solution for 60 min. In the group AP, reperfusion based on the I/R model was immediately followed by infusion of K-H solution containing the ASIC3 blocker APETx2 (630 nM, 5 min, Abcam, United Kingdom). APETx2 is a specific blocker of ASIC3 (Diochot et al., 2004). This was followed by continuous perfusion with K-H solution for an additional 55 min. Cardiac function parameters were continuously recorded, including heart rate (HR), left ventricular developed pressure (LVDP), left ventricular end-diastolic pressure (LVEDP), and the maximum rate of pressure increase/decrease in the left ventricle (+dp/-dtmax). At the end of reperfusion, ventricular tissue was collected and stored at −80°C.

### Myocardial Infarct Size Assessment

At the end of the experiment, the heart was quickly excised from the aortic root and placed in a pre-chilled PBS buffer to remove residual blood. It was sliced into five uniform sections from apex to base and incubated in freshly prepared 1% Evans Blue/2,3,5-triphenyl tetrazolium chloride (TTC) staining solution at 37°C for 20 min. The slices were then fixed in a specimen fixation solution for 24 h. Images were captured with a camera, and myocardial infarct size was analyzed using ImageJ software (Wang et al., 2021).

### ASIC3 Protein Extraction and Western blot Analysis

Left ventricular tissue (100 mg) from rats was collected and homogenized in RIPA lysis buffer with 2 μL of protease inhibitor. The samples were sonicated and centrifuged at 13,000 rpm for 10 min at 4°C. Protein quantification was conducted using the BCA method, and samples were stored at −80°C. Each sample was loaded with 30–50 μg of protein. Proteins were transferred to a membrane after SDS-PAGE separation. The membrane was washed three times with TBST on a shaker for 5 min each time and then blocked for 2 h. The membrane was incubated overnight at 4°C with an ASIC3 antibody (1:1,000, Abcam, United Kingdom) and a β-actin antibody (1:2,000, Abcam, United Kingdom). The following day, the membrane was washed three times with TBST. The membrane was then incubated with HRP-conjugated goat anti-rabbit IgG polyclonal antibody (1:20,000, HUABIO, China) and washed three times with TBST. ECL substrate was added and incubated for 5 min. The membrane was then exposed to detect protein bands, which were analyzed using ImageJ software.

### RNA Extraction and RT-qPCR Analysis

Left ventricular tissue (100 mg) from rats was collected, and total RNA was extracted using the Takara 9,108 kit. The extraction steps included lysis, phase separation, RNA precipitation, RNA washing, and RNA dissolution. Primers for ASIC3 and β-actin genes were designed based on GenBank sequences. RNA was reverse-transcribed into cDNA using the Takara RR047A kit (reaction conditions: 37°C for 15 min, 85°C for 5 s). RT-qPCR was performed using the RR820A kit and specific primers according to the manufacturer’s instructions (reaction conditions: 95°C for 0.5 min for 1 cycle; 95°C for 5 s, 60°C for 34 s for 40 cycles; 95°C for 15 s, 60°C for 1 min, 95°C for 15 s for 1 cycle).

### ASIC3 primer

Asic3-F GAC​TAT​CTC​TGT​GAG​GTT​TTC​CA.

Asic3-R CCA​TTC​AAC​TCT​TCC​TGG​AGC.

### β-actin primer

Actb-F AGG​GAA​ATC​GTG​CGT​GAC​AT.

Actb-R TGCCGATAGTGATGACCTGA.

### Enzyme-linked immunosorbent assay (ELISA)

Coronary perfusate and left ventricular tissue stored at −80°C were retrieved. The tissue was homogenized using an ultrasonic homogenizer and centrifuged at 5,000 g for 5–10 min to obtain the supernatant. The coronary effluent thawed on ice was directly centrifuged to obtain the supernatant. According to the ELISA kit instructions (Jianglai Biology, Shanghai), different concentrations of standards were added to the standard wells. 50 μL of sample solution was added to the sample wells, followed by 100 μL of HRP-conjugated antibody in each well. The plate was incubated at 37°C for 60 min and washed five times. 50 μL of Substrate A and 50 μL of Substrate B were added to each well and incubated in the dark at 37°C for 15 min. After adding the stop solution, CK-MB, LDH, MDA, and SOD levels were immediately measured. The standard curve was plotted according to the ELISA kit instructions, and the experimental results were calculated (Xia et al., 2022).

### ATP levels were measured using a luminometer

Left ventricular tissue from rats was lysed in lysis buffer and homogenized using a glass homogenizer. The standard curve was generated according to the kit instructions (Beyotime Biotechnology, China). After preparing the ATP working solution, 100 μL of the ATP detection reagent was added to each well and incubated at room temperature for 3–5 min. Then, 20 μL of sample or standard was added to each well, mixed quickly, and the RLU value was immediately measured. The ATP concentration in the samples was calculated using the standard curve (Previs et al., 2022).

### Transmission electron microscopy (TEM)

At the end of the experiment, hearts were quickly excised and placed in a pre-chilled PBS buffer to remove residual blood. Left ventricular tissue was embedded and fixed for TEM observation of myocardial ultrastructure. Mitochondrial damage was assessed using the Flameng scoring system (Lam et al., 2021).

### Metabonomics analysis

Six myocardial tissue samples (25 mg each) were collected from each group and mixed with ethanol and acetonitrile. After sonication for 10 min, the samples were centrifuged at 25,000 rpm for 15 min. The supernatant was transferred to LC-MS vials and stored at −80°C. Quality control (QC) samples were prepared by mixing equal volumes of supernatant from all samples. Primary and secondary mass spectrometry data were collected using a Q Exactive mass spectrometer (Thermo Fisher Scientific, United States). QC samples were used to evaluate method stability. Raw mass spectrometry data were imported into Compound Discoverer 3.1 (Thermo Fisher Scientific, United States) for initial processing and then into XCMS (version 3.2) for data preprocessing. The preprocessed data were analyzed using multivariate statistical analysis.

Differential metabolites were identified using orthogonal partial least squares discriminant analysis (OPLS-DA), univariate analysis (fold change, FC), and Student’s t-test. Final identification of differential metabolites between groups was based on a VIP score ≥1 from the OPLS-DA model, fold change ≥1.2 or ≤0.85, and a p-value <0.05^31^. Additionally, Pearson correlation analysis was performed to correlate differential metabolites with cardiac function in experimental rats. Pathway functional annotation was conducted using the KEGG (Kyoto Encyclopedia of Genes and Genomes) database to identify the main biochemical metabolic and signal transduction pathways involved.

### Statistical analysis

Statistical analyses were performed using GraphPad Prism (Version 10.1.2) and SPSS (Version 24.0). Normally distributed data are presented as mean ± SD. Groups were compared using Student’s t-test for normally distributed data. Hemodynamic parameters at the same time point in different groups were compared using two-way ANOVA followed by Sidak’s multiple comparisons test. Comparisons of the Flameng scores among different groups were conducted using the Kruskal-Wallis test, followed by Dunn’s *post hoc* test for multiple comparisons. Stepwise multivariate linear regression and Pearson’s correlation analysis were used to identify correlations between metabolite levels and LVDP, using MetaboAnalyst 4.0. All data were derived from at least of six independent experiments, with specific information for each experiment detailed in the relevant figure legend. Differences were considered significant at *P < 0.05; **P < 0.01; ***P < 0.001; ****P < 0.0001.

## Result

### APETx2 post-conditioning improved hemodynamics in MIRI hearts

Before myocardial ischemia, there were no statistical differences in HR, LVDP, LVEDP, and ±dp/dtmax among the three groups (Figures 1B–F). As the heart resumed beating and the duration of myocardial reperfusion increased, cardiac function in all groups showed a decreased trend. Compared to the group Nor, the I/R group showed a more pronounced decrease in HR, LVDP, and ±dp/dtmax, and a significant increase in LVEDP. The group AP showed improvements in all cardiac function indicators compared to the group I/R. Notably, the improvement in LVEDP was particularly pronounced in the group AP (Figures 1B–F). These results indicate that APETx2 post-conditioning improved I/R-induced hemodynamic dysfunction.

### APETx2 post-conditioning inhibited ASIC3 expression and reduced myocardial infarct size

**FIGURE 2:**
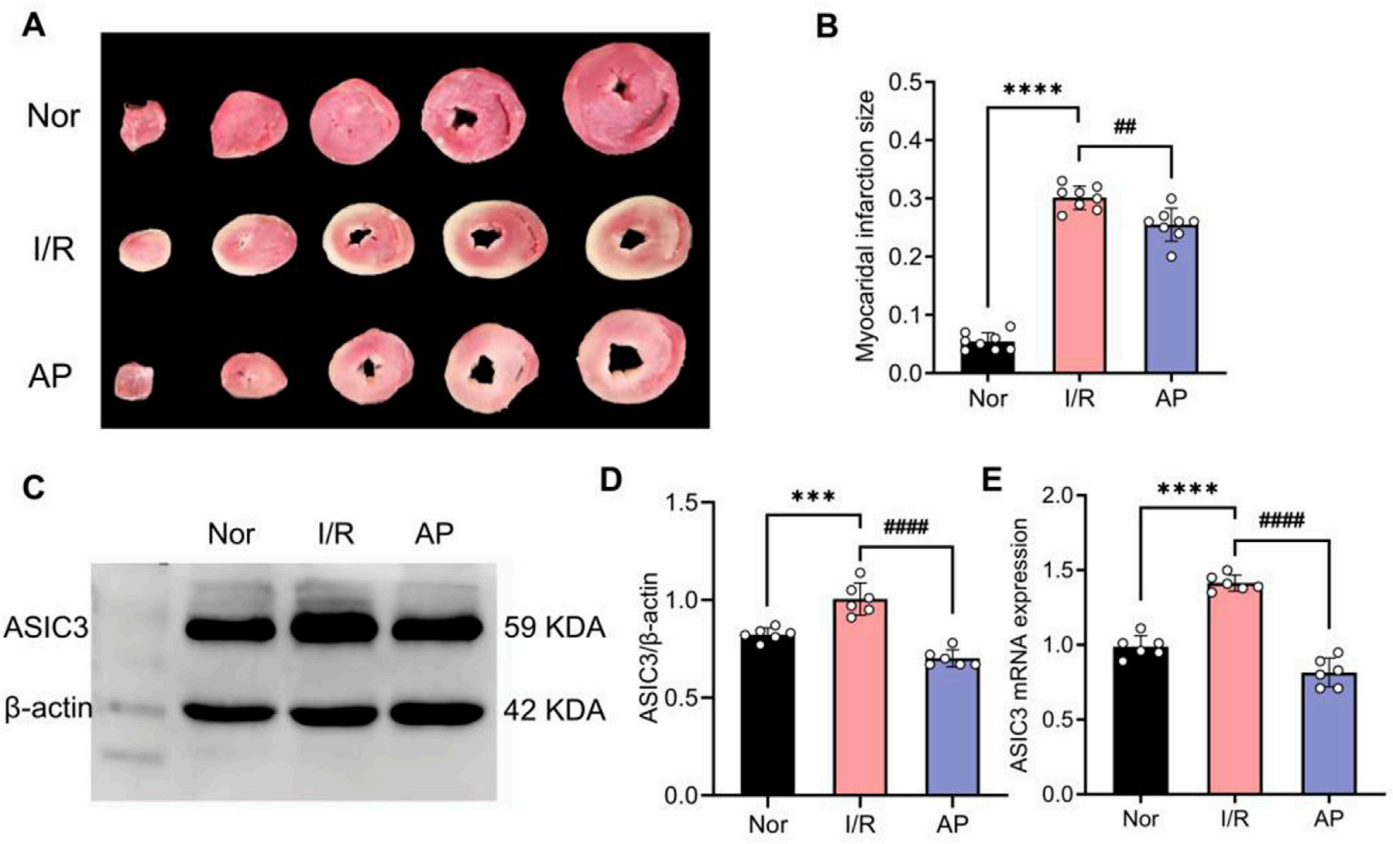
APETx2 post-conditioning inhibits ASIC3 expression and reduces myocardial infarct size. **(A)** Myocardial infarct size was observed using TTC staining. Compared to the Nor group, myocardial infarct size significantly increased in the I/R group after MIRI, whereas APETx2 post-conditioning significantly reduced the infarct size (n = 3 per group). **(B)** Quantification of myocardial infarct size. Infarct size was calculated using ImageJ software. **(C)** ASIC3 protein expression in rat myocardial tissue. Post-conditioning with the ASIC3 antagonist APETX2 (630 nM) inhibited ASIC3 protein expression in MIRI rats (n = 6 per group). **(D)** Quantification of ASIC3 protein expression. **(E)** ASIC3 mRNA expression in rat myocardial tissue. APETx2 inhibited ASIC3 mRNA expression in MIRI rats (n = 6 per group). One-way ANOVA, *P < 0.05; **P < 0.01; ***P < 0.001; ****P < 0.0001. All data are presented as mean ± SEM

The experiment showed that myocardial infarct size significantly increased in group I/R compared to the Nor group in isolated rat hearts. The myocardial infarct size in group AP was lower than that in the I/R group (Figures 2A, B). Additionally, the APETx2 inhibitor significantly suppressed myocardial ASIC3 expression (Figures 2C–E). This finding suggests an ASIC3 inhibitor can reduce myocardial infarct size in isolated rat hearts.

### APETx2 post-conditioning ameliorated myocardial cell injury

**FIGURE 3:**
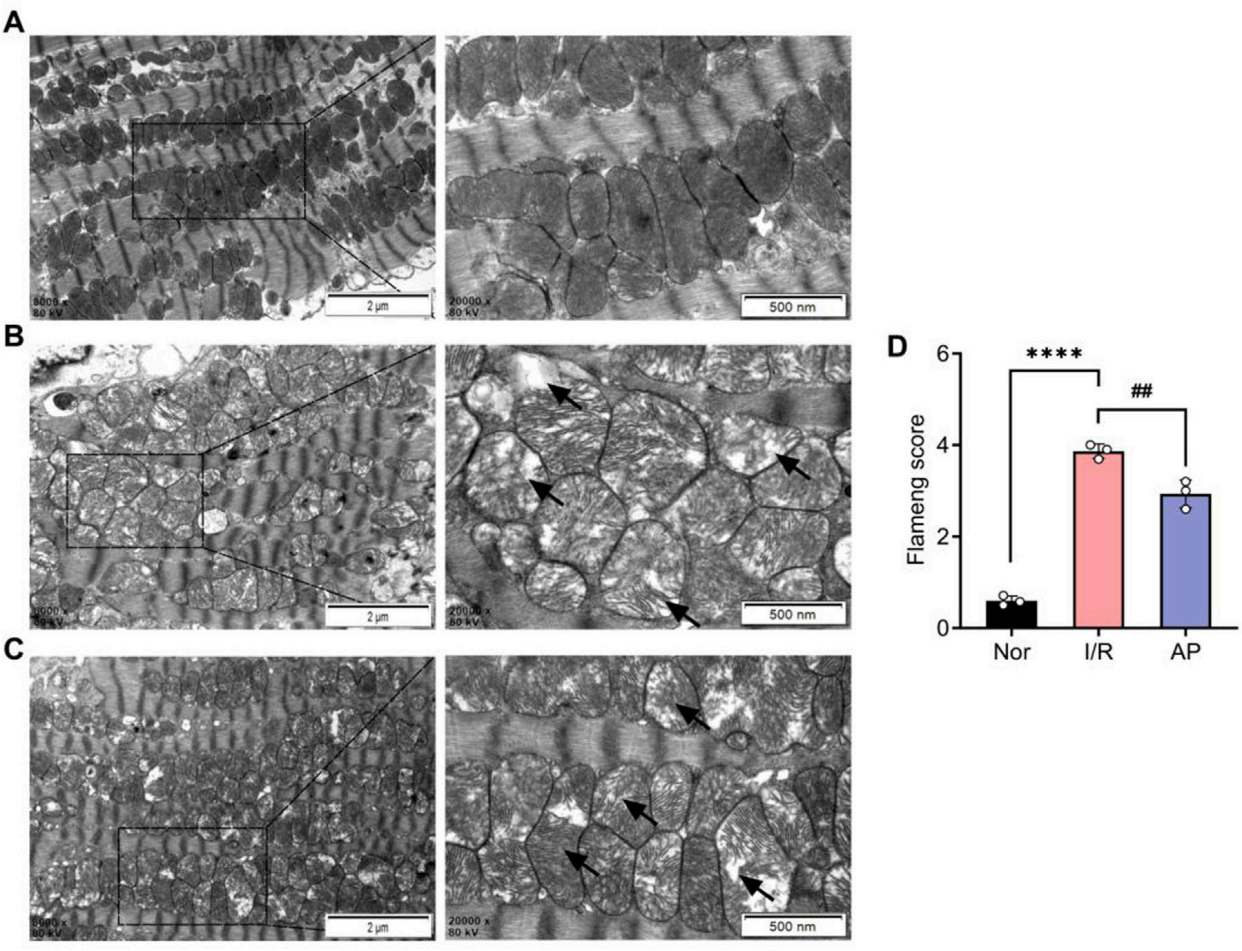
APETx2 post-conditioning improved myocardial cell injury. **(A)** Ultrastructure of the group Nor myocardial cells under transmission electron microscopy. The morphology of myocardial cells was normal, with oval-shaped mitochondria arranged neatly and uniformly electron-dense. Myofibrils were orderly arranged, with clearly defined dark and light bands, and distinct Z-lines and M-lines (n = 3 per group). **(B)** Ultrastructure of the group I/R myocardial cells under transmission electron microscopy. The morphology of myocardial cells was significantly abnormal. Many mitochondria were markedly swollen, with reduced or fractured cristae, sparse or vacuolated matrix, and decreased electron density. Myofibrils were disorganized, with indistinct or absent Z-lines and M-lines. Dark and light bands were not distinguishable. Autophagy was observed in the cytoplasm (magnification: X8000, X20000, n = 3 per group). **(C)** Ultrastructure of the group AP myocardial cells under transmission electron microscopy. The morphology of myocardial cells showed some recovery compared to the group I/R. Mitochondrial swelling was reduced, with increased volume, fewer cristae fractures, and slight matrix dissolution, but decreased electron density. Myofibrils were relatively disorganized, with indistinct Z-lines and M-lines. Autophagy was observed in the cytoplasm (magnification: X8000, X20000, n = 3 per group). **(D)** Flameng score (n = 3 per group). Flameng scores were lower in the group I/R than in the I/R group Nor (P < 0.0001). Flameng scores were higher in the group AP than in the group (P < 0.01), suggesting that APETx2 post-conditioning can improve myocardial mitochondrial injury. Two-way ANOVA, *P < 0.05; **P < 0.01; ***P < 0.001; ****P < 0.0001. All data are presented as mean ± SEM.

We used transmission electron microscopy to observe cardiomyocyte ultrastructure among different groups (Figures 3A–C). The myocardial cells in group I/R exhibited significant morphological abnormalities, including marked mitochondrial swelling, reduced or fractured cristae, and sparse, dissolved, or vacuolated matrix. Myofibrils were disorganized with indistinct or missing Z-lines and M-lines. A small amount of autophagy was also observed in the cytoplasm (Figure 3B). Myocardial cell morphology partially recovered after APETx2 post-conditioning. This was evidenced by reduced mitochondrial swelling, increased volume, fewer cristae fractures, and less myofibril disorganization. A small amount of autophagy was still observed in the cytoplasm (Figure 3C). The Flameng score for mitochondrial damage in the group AP was significantly lower than that in the group I/R (p < 0.01) (Figure 3D).

### APETx2 post-conditioning mitigated myocardial injury and oxidative stress

**FIGURE 4:**
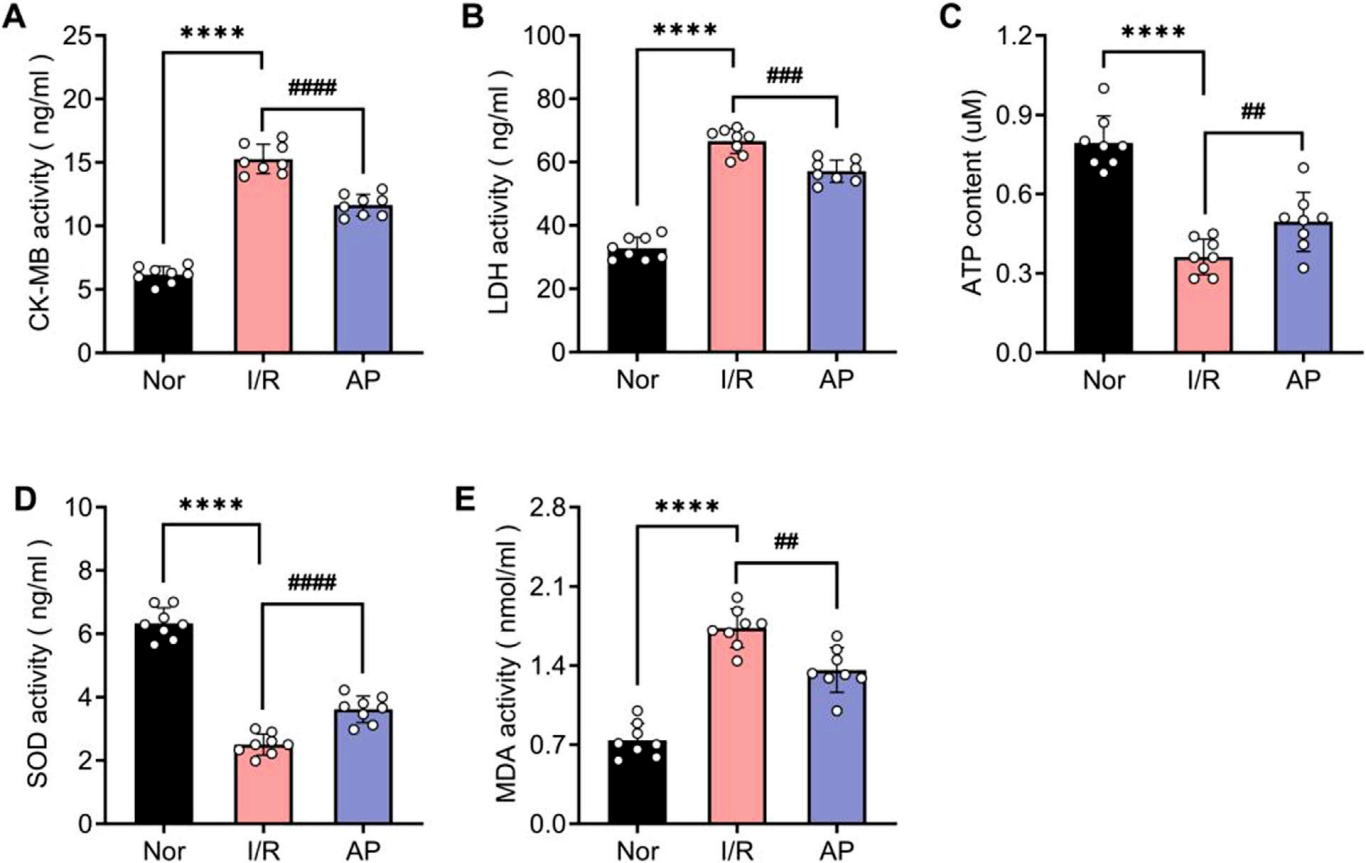
APETx2 post-conditioning improves I/R-induced myocardial injury and oxidative stress. Nor **(A, B)** CK-MB and LDH levels in coronary perfusate were collected after 30 minutes of reperfusion. CK-MB and LDH levels were significantly increased in the group I/R compared to the group and were significantly reduced by APETx2 post-conditioning (n = 8 per group). **(C)** ATP content. ATP content in myocardial tissue was significantly lower in the I/R group than in the group Nor, and this decrease was significantly attenuated by APETx2 post-conditioning (n = 8 per group). **(D, E)** SOD and MDA levels in coronary perfusate were collected after 30 minutes of reperfusion. SOD levels were significantly decreased, and MDA levels were significantly increased in the I/R compared to the group Nor. APETx2 post-conditioning reversed these changes (n = 8 per group). One-way ANOVA, *P < 0.05; **P < 0.01; ***P < 0.001; ****P < 0.0001. All data are presented as mean ± SEM.

To further confirm the molecular mechanism by which APETx2 post-conditioning ameliorates MIRI, we used ELISA to examine markers of myocardial injury and indicators of oxidative stress among different groups. The coronary perfusate was collected from isolated rat hearts after 30 min of reperfusion. ELISA results revealed that MIRI significantly elevated myocardial injury markers CK-MB and LDH, whereas APETx2 post-conditioning attenuated myocardial injury (Figures 4A, B). Additionally, ATP luminometer analysis indicated a significant decrease in energy supply in myocardial tissue following MIRI, which was improved by APETx2 post-conditioning (Figure 4C). Finally, ELISA results showed that MIRI caused abnormalities in myocardial oxidative stress markers SOD and MDA, which were alleviated by APETx2 post-conditioning (Figures 4D, E).

### Multivariate statistical analysis

**FIGURE 5:**
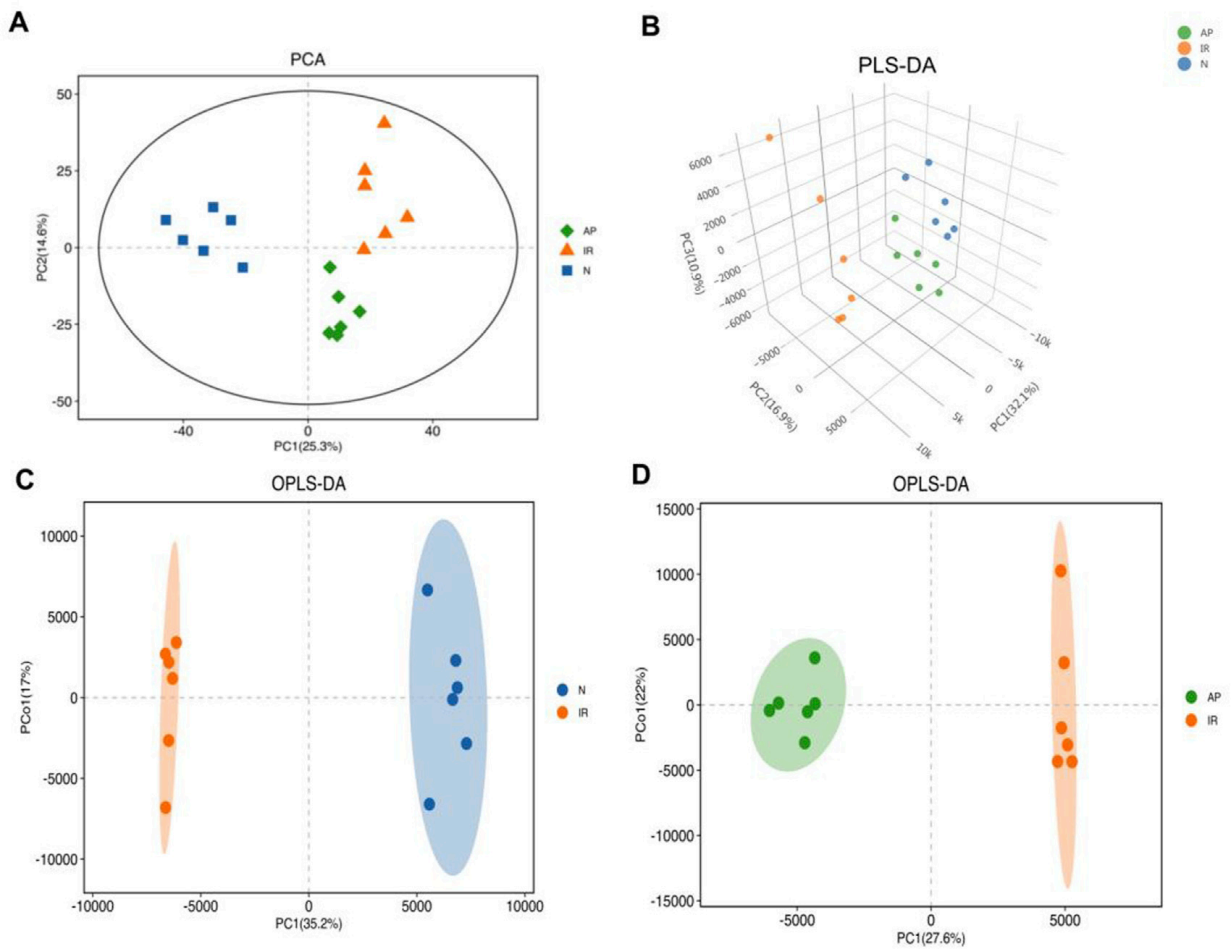
Multivariate statistical analysis. **(A)** PCA score plot. Metabolite profiles of each group exhibited clustering with clear separation, indicating differences in metabolic profiles among the three groups. **(B)** PLS-DA score 3D plot. This supervised statistical method shows significantly differentiates in metabolic profiles among the three groups. **(C, D)** OPLS-DA score plots. These plots, based on PLS-DA, enhance the model's interpretive power and effectiveness, indicating significant differences in metabolites between the I/R vs Nor and AP vs I/R groups.

OPLS-DA was performed based on PLS-DA to filter out noise unrelated to classification information, enhancing the model’s interpretive power and effectiveness. OPLS-DA provides more reliable data interpretation. Evaluation parameters for the OPLS-DA model include R^2^Y and Q2, representing the model’s explanatory power and predictive ability for the Y matrix, respectively (Shastry and Dunham-Snary, 2023). The parameters were: R^2^Y = 0.99, Q2 = 0.897 (Nor group vs. I/R group); R^2^Y = 0.994, Q2 = 0.954 (I/R group vs. AP group). The score plots showed that each group was well-separated, indicating that the relevant parameters can be predict accurately. This study used the OPLS-DA model to screen for differential metabolites, revealing significant differences among the groups (Figures 5A–D).

### Identification of metabolites

**FIGURE 6:**
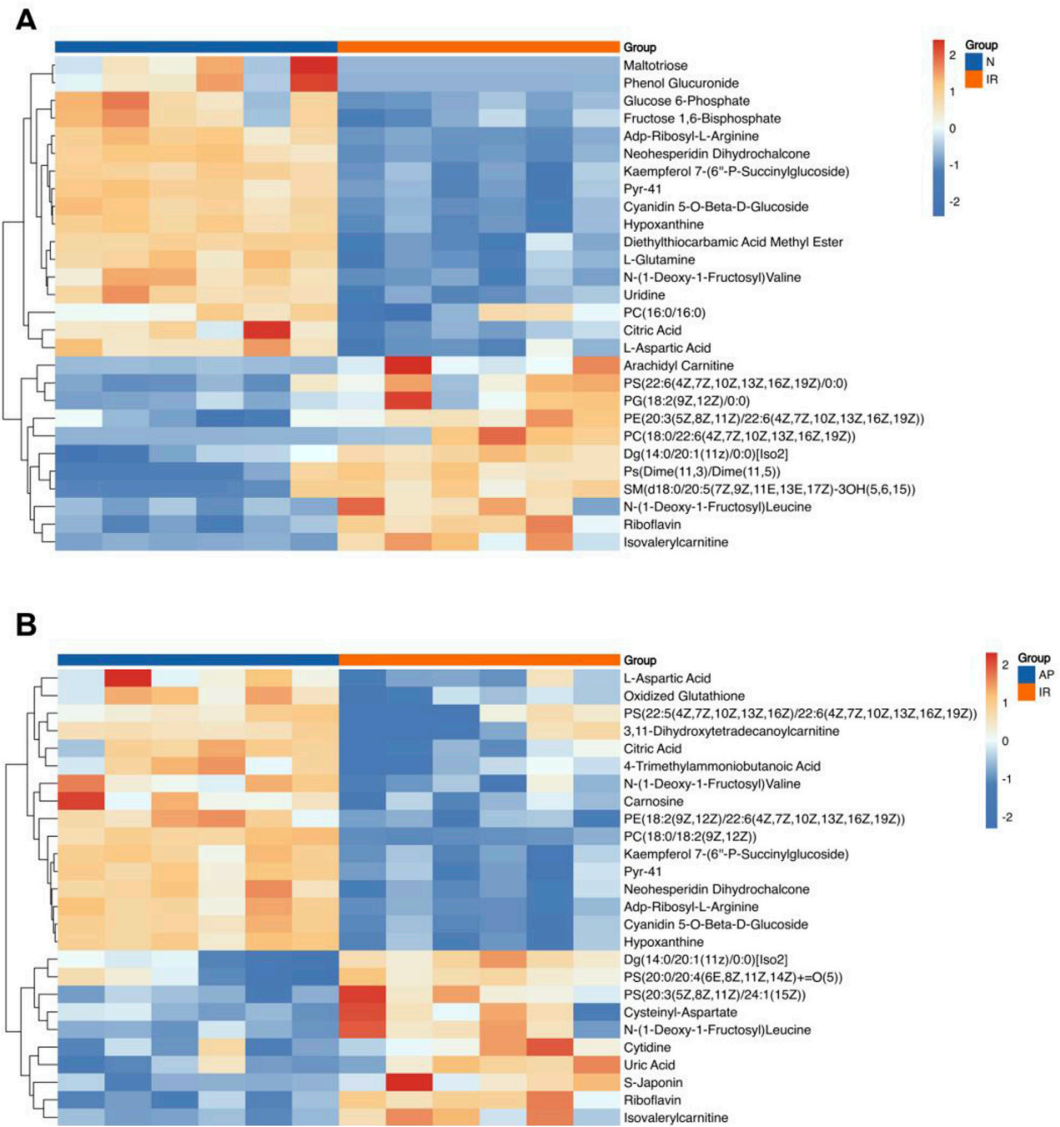
Heatmap of differential metabolites. **(A)** Heatmap of differential metabolites for I/R vs Nor. **(B)** Heatmap of differential metabolites for AP vs I/R. The horizontal axis represents the sample groups, and the vertical axis represents the metabolites. Warmer colors indicate higher expression levels, while cooler colors indicate lower expression levels (n = 8 per group).

This study conducted a metabolomic analysis on tissues subjected to MIRI and treated with the ASIC3 inhibitor APETx2. After merging, de-duplicating, and screening for functional metabolites, we found that 17 metabolites were significantly upregulated and 11 were significantly downregulated considerably following myocardial ischemia/reperfusion injury (Figure 6A). These metabolites may reflect changes in endogenous metabolites in the isolated rat heart following MIRI. Compared to the I/R group, APETx2 post-conditioning resulted in the significant upregulation of 16 metabolites and the considerable downregulation of 10 metabolites (Figure 6B). These differential metabolites may be related to ASIC3 channels.

### Six metabolites are relevant to APETx2 post-conditioning

**FIGURE 7:**
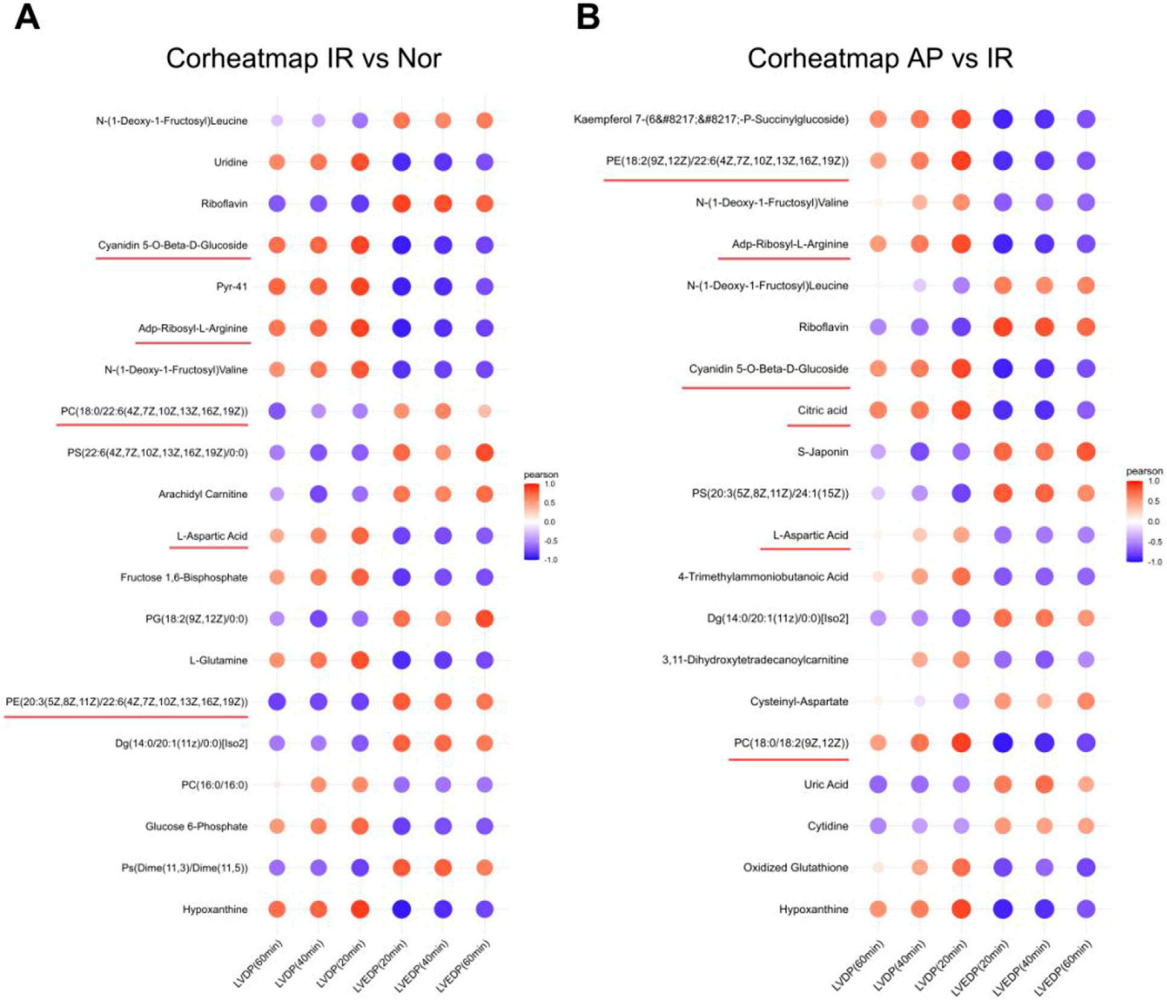
Heatmap of correlation analysis between selected metabolites and cardiac function in experimental rats. **(A)** Correlation analysis between metabolites and cardiac function in I/R vs Nor groups (n = 8 per group). **(B)** Correlation analysis between metabolites and cardiac function in AP vs I/R groups (n = 8 per group). The color represents the Pearson correlation coefficient.

Further analysis was performed on the initially screened differential metabolites, correlating them with cardiac function (LVDP, LVEDP) in experimental rats. Based on the Pearson coefficient and research context, the metabolites associated with APETx2 post-conditioning were identified: Phosphatidylcholine (PC) (P = 0.0035), Phosphatidylethanolamine (PE) (P = 0.0137), ADP-ribosyl-L-arginine (P = 0.000245), cyanidin 5-O-beta-D-glucoside (P = 0.008), citric acid (P = 0.0092) and L-aspartic acid (P = 0.0051) (Figures 7A, B).

### Biological pathway analysis 

**FIGURE 8:**
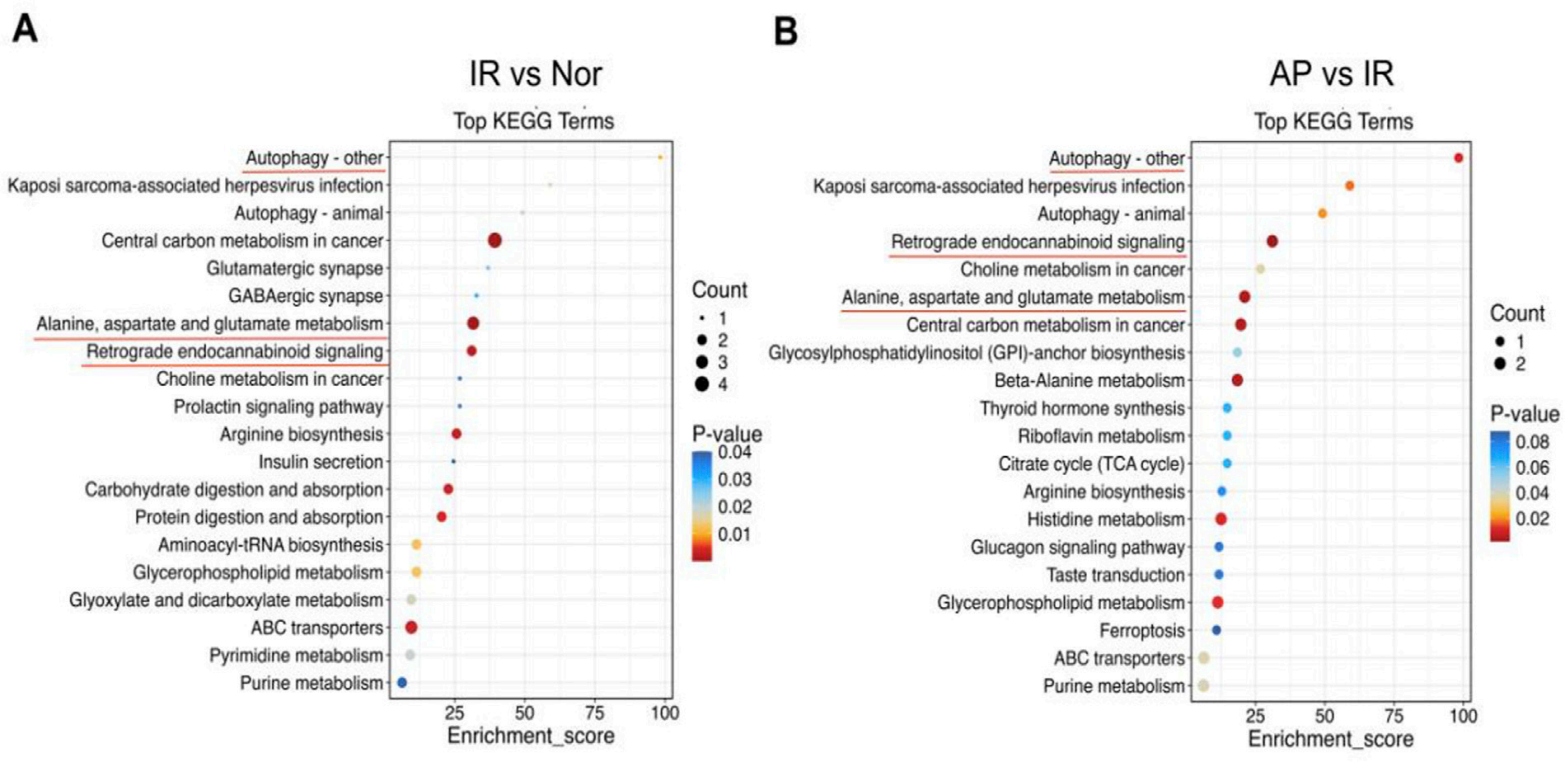
KEGG pathway analysis. **(A, B)** KEGG pathway enrichment bubble chart. From panels **(A, B)**, the *X*-axis represents the enrichment ratio, and the *Y*-axis represents the KEGG pathway. The bubble size represents the number of genes annotated to the KEGG pathway. The color represents the enrichment significance (n = 6 in each group).

Pathway analysis was conducted to investigate the biological functions of the altered metabolites. KEGG enrichment analysis revealed that multiple metabolic pathways were involved in the APETx2 post-conditioning process of MIRI, including autophagy, endocannabinoid signaling, alanine, aspartate, and glutamate metabolism (Figures 8A, B).

## Discussion

In this study, we found that APETx2 post-conditioning significantly alleviated myocardial ischemia-reperfusion injury. APETx2 post-conditioning enhanced cardiac hemodynamics, reduced myocardial infarct size and lessened myocardial cell damage. It also improved myocardial injury markers and oxidative stress indicators. Additionally, this study found that MIRI caused multiple metabolic changes. PC, PE, ADP-ribosyl-L-arginine, citric acid, cyanidin 5-O-beta-D-glucoside, and L-aspartic acid decreased after MIRI. However, APETx2 post-conditioning partially restored these metabolite levels. These metabolites are primarily associated with endocannabinoid signaling and autophagy pathways. These findings suggest that these differential metabolites exert cardioprotective effects primarily through the autophagy pathway and endogenous cannabinoid signaling pathway.

The ASIC family, ASIC3, is widely but non-specifically distributed in the central and peripheral nervous systems (Yagi et al., 2006; Dulai et al., 2021). Tomonori Hattori demonstrated that ASIC channels are highly expressed in the Dorsal Root Ganglion(DRG) (Hattori et al., 2009). ASIC3 is the predominant subtype in cardiac DRG. ASIC3 functions as a sensitive pH and lactate sensor during myocardial ischemia, and its activation can trigger harmful neuronal reflexes (Dulai et al., 2021; Hattori et al., 2009). Junichi Yagi found that most cardiac sensory neurons contain ASIC3. ASIC3 generates sustained currents within the extracellular pH range of 7.3 to 6.7 during myocardial ischemia in rats (Yagi et al., 2006). Our study also found that ASIC3 is present in the myocardium and abundantly expressed. Wu (Shudong et al., 2015) discovered that downregulating the expression of ASIC2 and ASIC3 genes in myocardial ischemic rats effectively mitigated myocardial cell injury by blocking the opening of ASICs.

APETx2 is a 42-amino-acid peptide crosslinked by three disulfide bridges, structurally similar to other sea anemone toxins that inhibit voltage-sensitive Na^+^ and K^+^ channels (Diochot et al., 2004; Jalalvand et al., 2016). The inhibition of ASIC3 currents was rapid and saturated within 30 s of APETx2 perfusion (Diochot et al., 2004). In in vitro experiments, APETx2’s inhibitory effect typically persists for several tens of minutes or longer before its potency gradually diminishes (Karczewski et al., 2010). APETx2 selectively inhibits the H^(+)^-gated sodium channel ASIC3 (Blanchard et al., 2012). This toxin does not inhibit ASIC1 isoforms (ASIC1a and ASIC1b) or ASIC2. It exhibits low affinity for ASIC1b-ASIC3 and ASIC1a-ASIC3 and no effect on ASIC2a-ASIC3 channels (Diochot et al., 2004). Regarding the ASIC2b-ASIC3 channel, although APETx2 shows inhibitory activity, minimal or absent ASIC2 expression in cardiac tissue allows for the exclusion of this effect in the current context (Redd et al., 2021). Furthermore, recent studies indicate that APETx2 also inhibits NaV1.8 channels, with an IC50 of approximately 2.6 µM^35^. In the present study, however, the concentration of APETx2 used was 630 nM, substantially below the IC50, suggesting a negligible effect on NaV1.8 channels. In conclusion, We selected APETx2, a specific inhibitor of ASIC3, to evaluate whether inhibiting ASIC3 has cardioprotective effects. Experiments demonstrated that inhibiting ASIC3 significantly improved cardiac function in MIRI rats, particularly in the recovery of LVEDP. Additionally, it improved the levels of markers such as LDH and CK-MB and decreased the extent of myocardial infarction. Similar results were observed in the ultrastructural examination of myocardial tissue. Furthermore, APETx2 exerted no significant effect on the rat heart under normal conditions (Supplementary Figure S1). These data further indicate that APETx2 post-conditioning is an essential protective mechanism in myocardial ischemia-reperfusion injury.

The metabolite alterations induced by the activation of ASIC3 channels may be a significant cause of myocardial damage (Ranjbarvaziri et al., 2021; McGarrah et al., 2018). Our screening identified several differential metabolites, with phospholipids constituting a substantial proportion. Furthermore, blocking the ASIC3 channel can partially restore the expression of metabolites such as PC and PE. These altered metabolites are primarily associated with autophagy, endocannabinoid signaling, and related pathways.

PE is an essential phospholipid crucial for biological development, mitochondrial function, and membrane structure, serving as the foundation for various biological pathways (Calzada et al., 2016). PE is abundantly synthesized via two major pathways: the CDP-ethanolamine pathway in the endoplasmic reticulum and the mitochondrial phosphatidylserine decarboxylase (PSD) pathway (Polyansky et al., 2022; Hsu and Shi, 2017). Among these pathways, the PSD pathway is crucia in maintaining mitochondrial morphology and function (Xu et al., 2022). PE is synthesized by phosphatidylserine decarboxylase 1 (PISD) following the translocation of phosphatidylserine (PS) to the mitochondria via the mitochondria-associated membrane (MAM) in the PSD pathway (Rockenfeller et al., 2015; Tasseva et al., 2013). Indeed, PE is associated with various cellular activities and functions, including autophagy and mitochondrial fusion (Polyansky et al., 2022; Tasseva et al., 2013). We observed a significant elevation of PE following APETx2 post-conditioning, suggesting its involvement in the autophagy process. The critical role of PE in the autophagy pathway has been well-documented in the literature (Rockenfeller et al., 2015; MacVicar et al., 2019; Zhao and Wang, 2020). Rockenfeller P. found that reducing intracellular PE levels following PISD knockdown accelerated ROS production and cell death. In contrast, it is significantly increasing intracellular PE levels by providing its precursor ethanolamine or through PISD overexpression markedly enhanced autophagic flux (Rockenfeller et al., 2015). In the present study, PE levels significantly decreased after ischemia/reperfusion (I/R), and APETx2 post-conditioning was able to reverse this reduction. We speculate that APETx2 post-conditioning might exert cardioprotective effects through PE-regulated autophagy.

PC is the predominant phospholipid in the outer leaflet of cell membranes, constituting a significant portion and serving as a vital component of biological membranes. (van der Veen et al., 2017). PC possesses antioxidant properties and can mitigate damage caused by elevated ROS in MIRI through a mechanism involving the reduction of methane, significantly decreasing inflammatory activation during I/R^47^ (Tratsiakovich et al., 2013b; Algoet et al., 2023). Cytidine-5′-phosphorylcholine (CDP-choline), an intermediate in PC synthesis, attenuates ischemia- and reperfusion-induced liver injury by reducing oxidative stress and preserving mitochondrial function (Yilmaz et al., 2008; d'Avanzo et al., 2024). Furthermore, decreased levels of PE, an intermediate in phospholipid synthesis, directly affect the downstream synthesis of PC (MacVicar et al., 2019). In the present study, levels of PC and PE decreased significantly following I/R. However, APETx2 post-conditioning modulated this process, which maybe involved in endogenous cannabinoid signaling (ECS). In our experiments, we observed significant elevations in PC and PE levels following APETx2 post-conditioning. Both are essential precursors for anandamide (AEA) synthesis in the ECS (van der Veen et al., 2017; Rezende et al., 2023). Therefore, we hypothesize that the cardioprotective effect of APETx2 post-conditioning might be related to the regulation of ECS intermediates and the maintenance of AEA function.

Cyanidin 5-O-beta-D-glucoside, commonly known as cyanidin, is an anthocyanin known for its antioxidant, anti-inflammatory, and neuroprotective effects, acting as an effective scavenger of oxygen radicals (Rahman et al., 2021; Mattioli et al., 2020). Pataki established a model of MIRI by administering anthocyanin via gastric gavage. They found that the incidence of ventricular fibrillation during reperfusion decreased by 42% compared to the model group, coronary flow rate increased by 32%, and ROS levels were reduced by 75% (Liobikas et al., 2016). Additionally, applying a PI3K inhibitor could completely block these effects, indicating that this signaling pathway is involved in the protective effect of cyanidin on acute myocardial ischemia-reperfusion injury (Zhang T. et al., 2023).

Aspartic acid and citric acid are prominent metabolic markers involved in amino acid metabolism throughout the pathophysiological process of MIRI (Zhang et al., 2024). L-aspartic acid acts is carrier for K^+^ and Mg^2+^ ions, delivering electrolytes to the myocardium and enhancing myocardial function (Aquilani et al., 2017). Additionally, the transamination of aspartic acid and α-ketoglutaric acid forms glutamate and oxaloacetate, which can enter the citric acid cycle (CAC) and increase substrate-level phosphorylation capacity (Olson et al., 2012). Citric acid is a crucial intermediate in the CAC and plays a significant role in inflammatory response-related pathways. It can mediate the AMPK signaling pathway, inhibit mTOR signaling, and is associated with several pro-inflammatory mediators, including NO, ROS, and prostaglandin E2 (PGE2) (Santos et al., 2023). Fei Mu found that citric acid levels decreased after MIRI, which is consistent with our findings (Mu et al., 2017). Our preliminary literature reported that post-conditioning with the mitoKATP channel opener, diazoxide increased the level of citric acid. These changes in metabolites may be involved in the myocardial protective mechanism of mitoKATP channel activation (Xiang et al., 2023). In the present study, levels of L-aspartate and citric acid were significantly downregulated after MIRI and notably restored following APETx2 post-treatment. These findings suggest that changes in these metabolites may be involved in the myocardial protective mechanisms of MIRI and the effects of APETx2 post-conditioning.

L-arginine is a conditionally essential amino acid, and its levels decrease following tissue damage and pathological changes (Tratsiakovich et al., 2013a; Bondonno et al., 2016). Studies have shown that ischemic brain injury leads to reduced arginine levels in blood and cerebrospinal fluid, with arginine levels negatively correlated with cerebral infarction and neurological impairment (Zheng et al., 2022). Other studies have reported that L-arginine may be an essential target for intervention in MIRI (Venardos et al., 2009; Cziráki et al., 2020). Adding L-arginine increases NO release, improves myocardial compliance after ischemia, and reduces coronary vascular resistance (Schreckenberg et al., 2015). Our results showed that L-arginine levels in the group AP were higher than those in the group I/R, and the specific mechanism underlying this observation requires further investigation.

## Study limitations

This study has several limitations. First, we should have included the classical ischemia-reperfusion model to verify the effects of APETx2 post-conditioning on MIRI and compare the results of different ischemia-reperfusion modules. Second, we did not perform *in vivo* experiments to confirm the metabolic changes and protective effects of APETx2 post-conditioning on MIRI. Finally, we did not validate the function of the identified metabolites or related pathways in MIRI. To address these shortcomings, we have conducted *in vitro* and *in vivo* experiments to validate the effects of ASIC3 KO on MIRI and associated pathways.

## Conclusion

In summary, this study indicates that APETx2 post-conditioning may alleviate MIRI, potentially through specific metabolic alterations, including modifications in PE and PC levels, as well as via pathways such as autophagy and endocannabinoid signaling. This study provides valuable data for future research on metabolism related to APETx2 post-conditioning and MIRI.

## Data Availability

The original contributions presented in the study are included in the article/Supplementary Material, further inquiries can be directed to the corresponding authors.
