# Supplementary figures and images for: Metabolomics study of APETx2 post-conditioning on myocardial ischemia-reperfusion injury
Source: Front Pharmacol. 2024 Dec 6;15:1470142. doi: 10.3389/fphar.2024.1470142 (PMC11658994; doi:10.3389/fphar.2024.1470142)

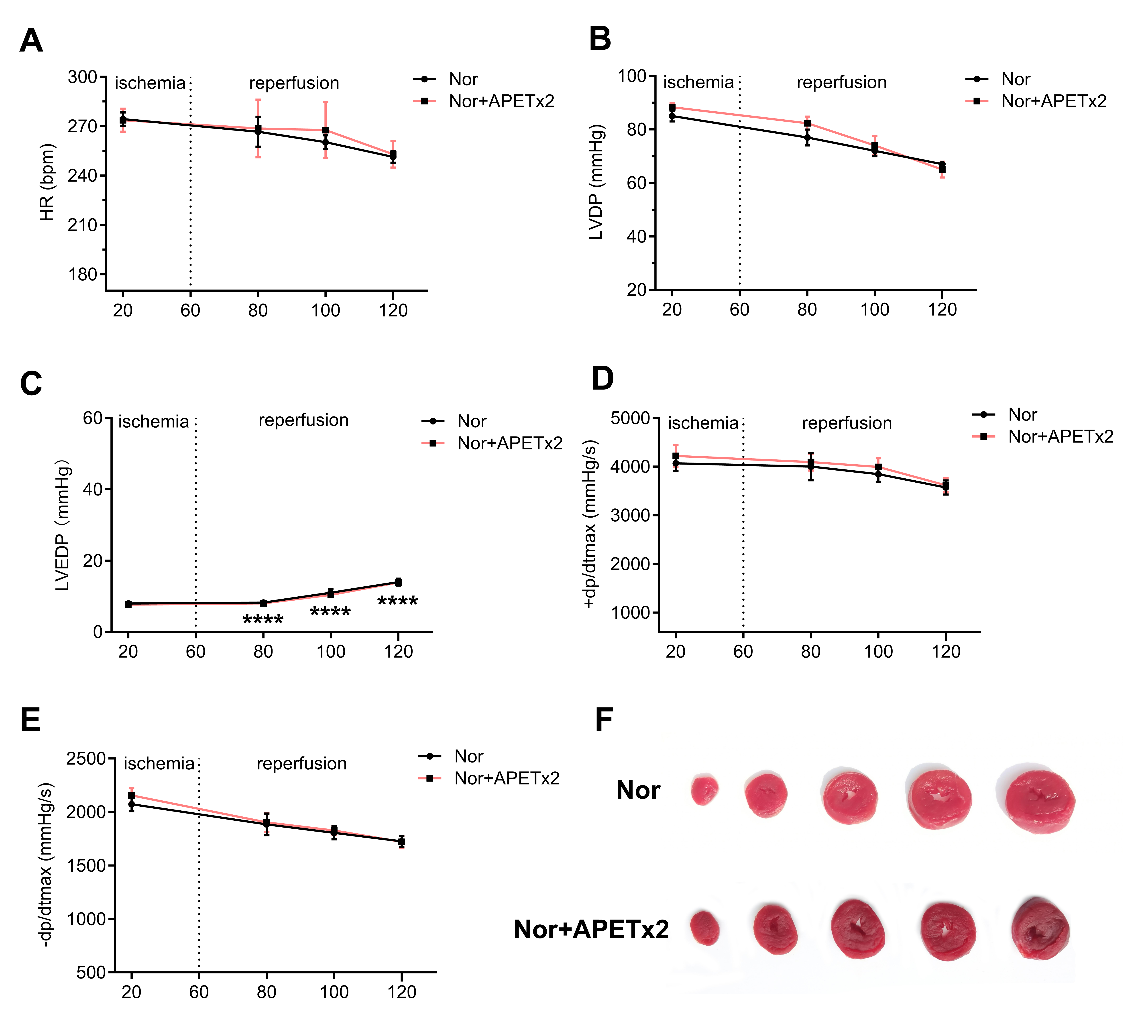

Supplement: Supplementary file 1 [file Image1.TIF]
